# Supplementary material for: Adhesion and Stability of Nanocellulose Coatings on Flat Polymer Films and Textiles
Source: Molecules. 2020 Jul 16;25(14):3238. doi: 10.3390/molecules25143238 (PMC7397189; doi:10.3390/molecules25143238)
Supplement: Supplementary file 1 [file molecules-25-03238-s001.pdf]

Supplementary Materials

Adhesion and Stability of Nanocellulose Coatings on Flat Polymer Films and Textiles

Raha Saremi [1,2], Nikolay Borodinov [3], Amine Mohamed Laradji [1], Suraj Sharma [1,2], Igor Luzinov [3], and Sergiy Minko [1,2,*]

[1] Nanostructured Materials Laboratory, University of Georgia, Athens, GA 30602, USA; raha@uga.edu (R.S.); alaradji@wustl.edu (A.M.L.); ssharma@uga.edu (S.S.)
[2] Department of Textiles, Merchandising and Interiors, the University of Georgia, Athens, GA 30602, USA
[3] Department of Materials Science and Engineering, Clemson University, Clemson, SC 29634, USA; nikolab@g.clemson.edu (N.B.); luzinov@clemson.edu (I.L.)
* Correspondence: sminko@uga.edu

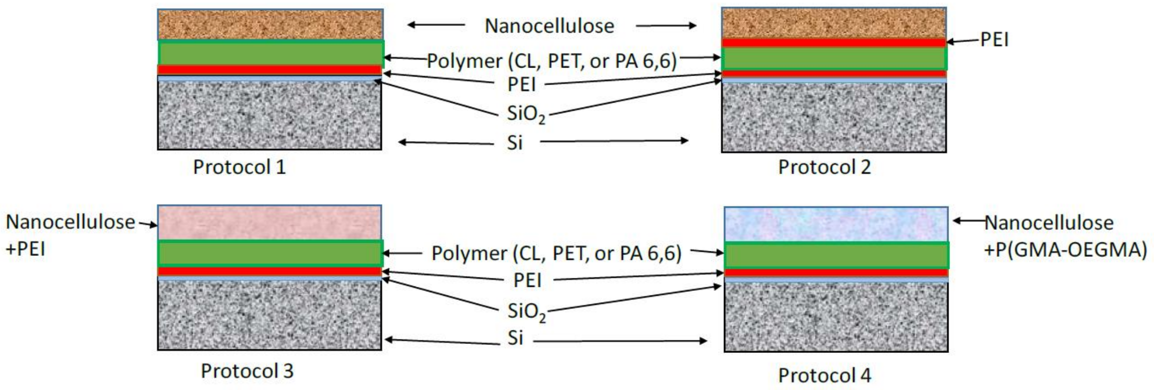

Figure S1. Schematics for the multilayered samples of nanocellulose coatings on the surface of polymer materials according to Protocols 1-4.

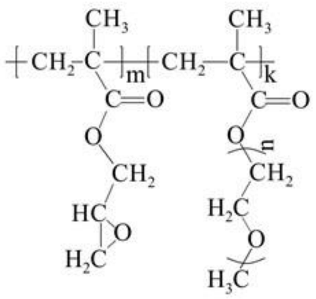

Figure S2. Structure of the P(GMA-OEGMA) copolymer

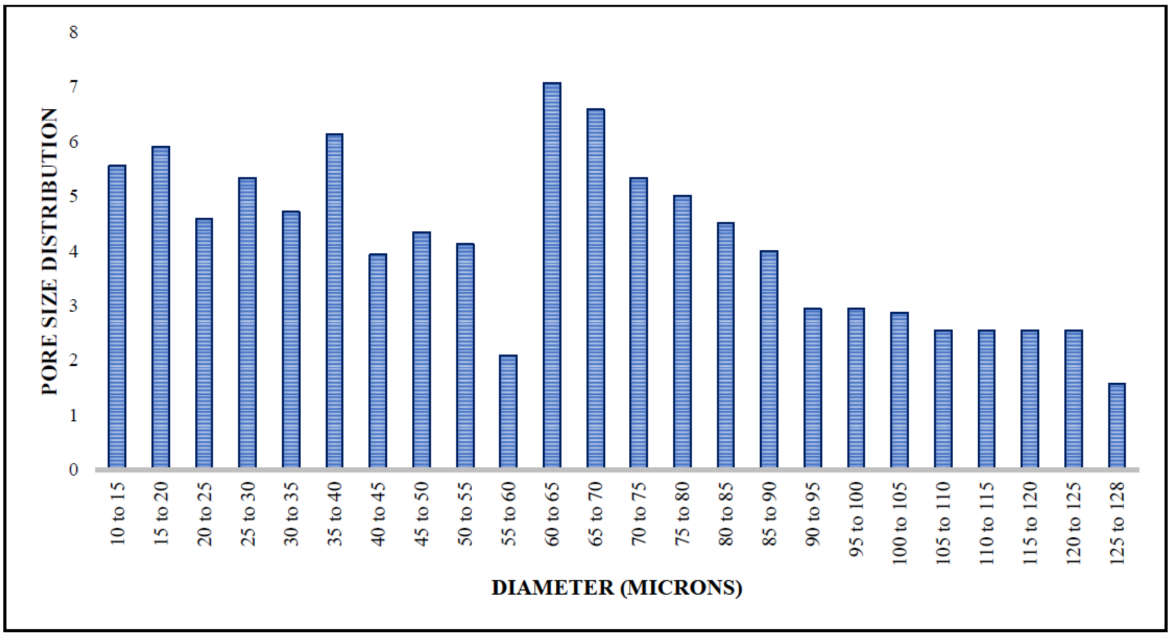

Figure S3. Pore size distribution of cotton fabrics

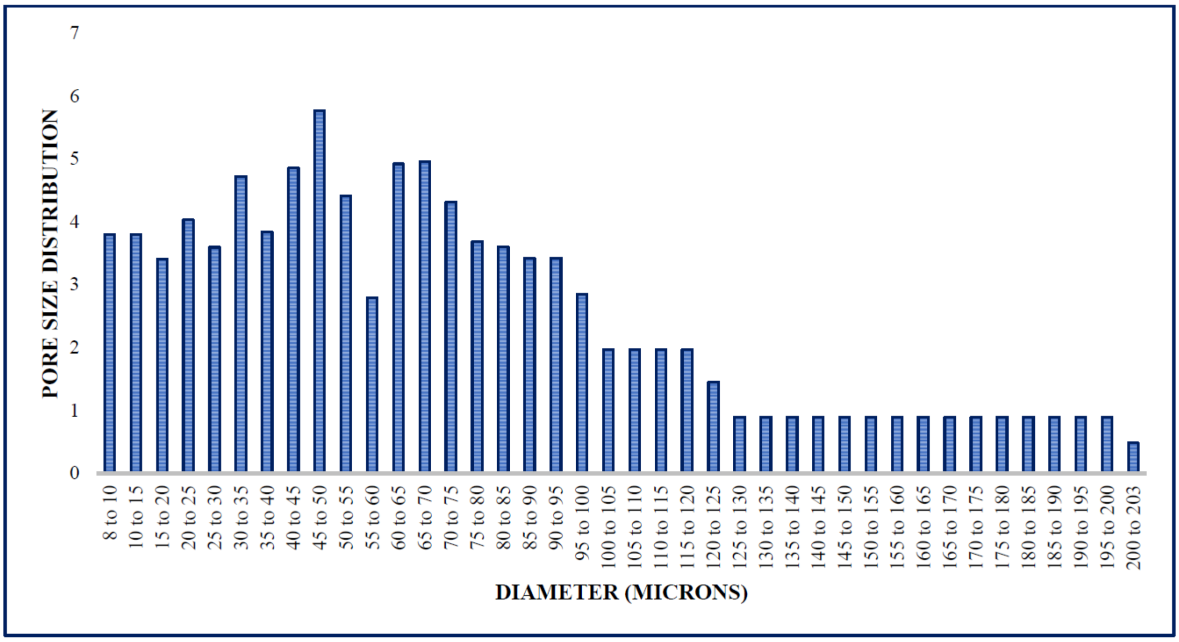

Figure S4. Pore size distribution of nylon fabrics

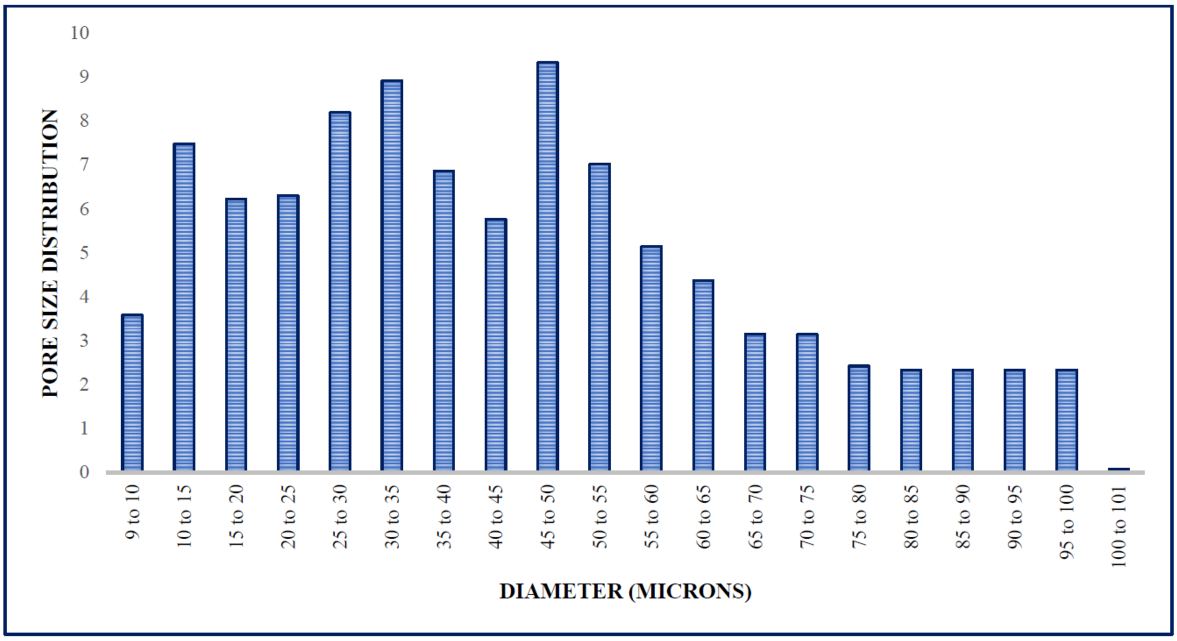

Figure S5. Pore size distribution of cotton/polyester fabrics

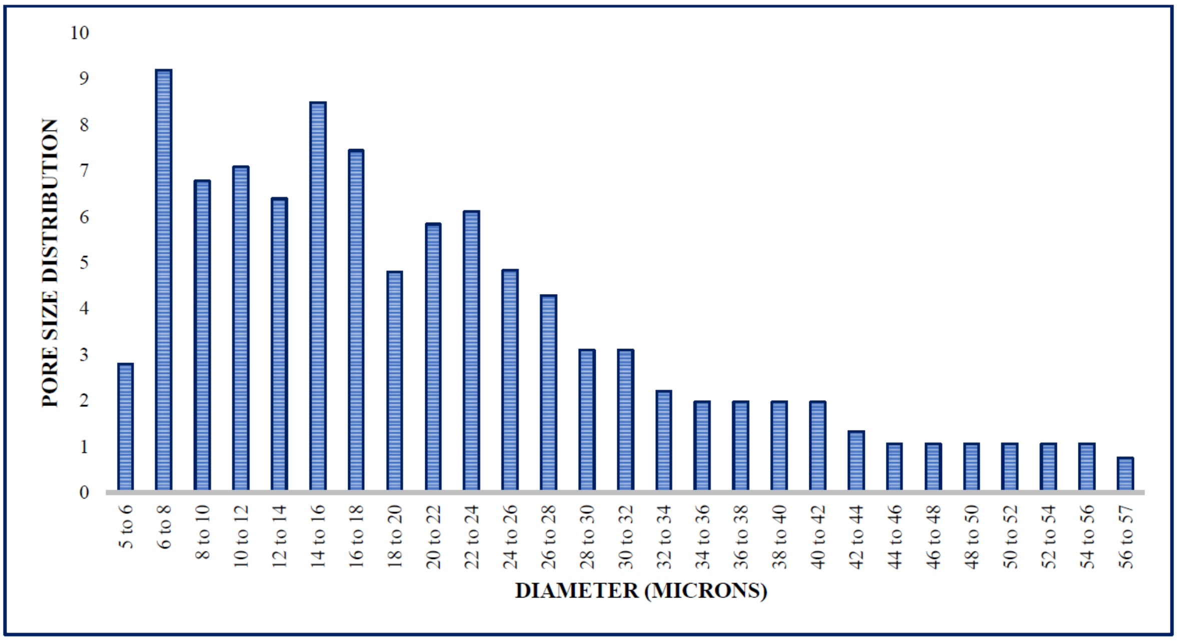

Figure S6. Pore size distribution of polyester fabrics

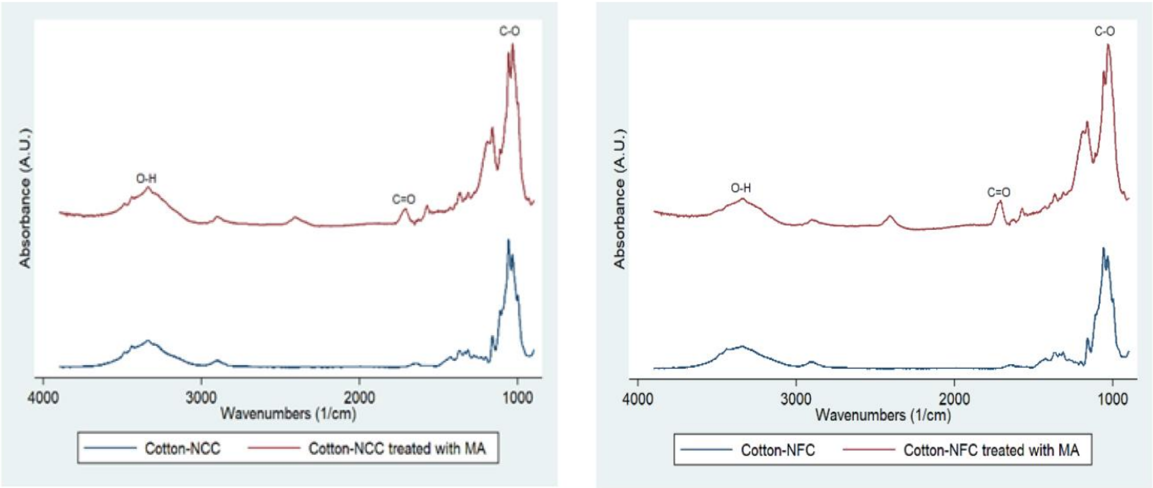

(a) (b)

Figure S7. FT-IR spectra of (a) cotton-NCC and cotton-NCC treated with MA and (b) cotton-NFC and cotton- NFC treated with MA

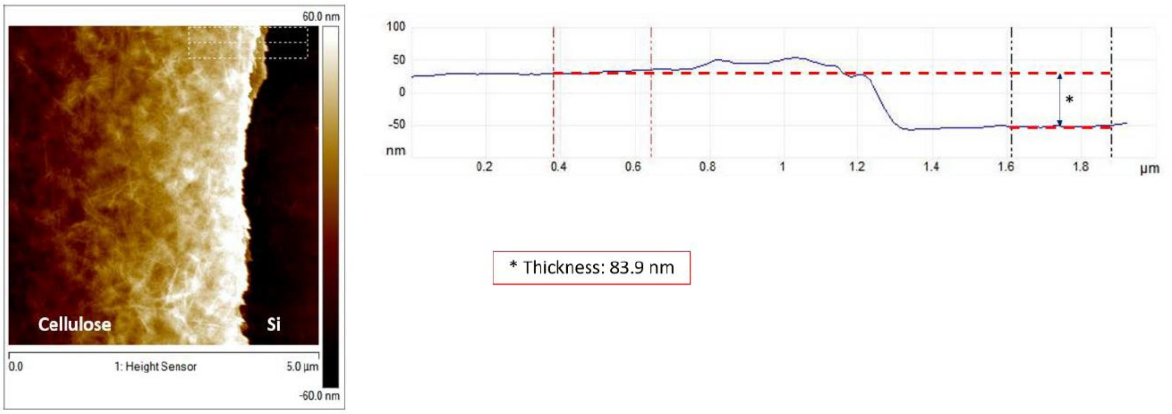

Figure S8. AFM topography image of the cellulose coating on the Si-wafer after the scratch with a steel needle

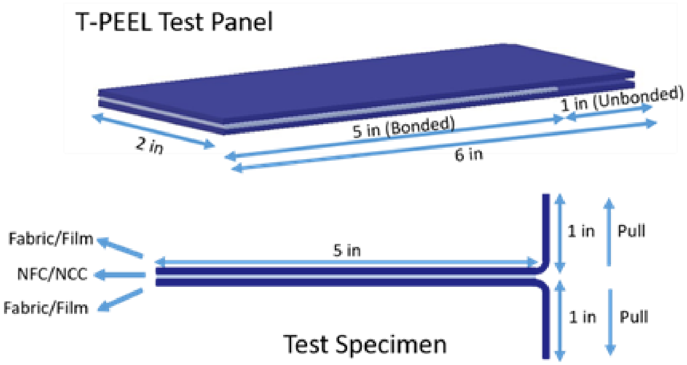

Figure S9. Schematic of a sample for the T-peel test
